# Between one event and two: The locus of the effect of stimulus contrast on temporal integration

**DOI:** 10.1111/psyp.13384

**Published:** 2019-04-29

**Authors:** Elkan G. Akyürek, Eria Wijnja

**Affiliations:** ^1^ Department of Psychology, Experimental Psychology University of Groningen Groningen The Netherlands

**Keywords:** attention, ERP, N1, N2pc, P3, stimulus contrast, temporal integration

## Abstract

The effects of relative stimulus contrast on temporal integration were investigated in a missing element task. Integration frequency was strongly modulated when the contrast of either the first or the second stimulus display was reduced. When the contrast of the first display was low, integration was enhanced, while it was reduced when the contrast of the second display was low. To reveal the processing phases implicated in these modulations of integration, the amplitude of ERP components was examined. N1 component amplitude was increased when the second display was low contrast, matching a full contrast condition. At the N2pc component, amplitude was strongly suppressed only in the former condition. P3 amplitude was also lowest when contrast on the second display was low but with successively increasing amplitudes observed for the other conditions, largely matching the pattern observed in behavioral performance. Taken together, contrast effects on temporal integration seem to originate from increased discriminative processing of the first stimulus display in particular (N1), which is consequently followed by an impairment in attentional processing (N2pc) and working memory consolidation (P3) of the missing element location.

## Abstract 2

Visual perception is often characterized in space, but in a dynamic world, time is equally important. The perceptual system tends to process relatively long‐lasting events in which information is accumulated through temporal integration. However, which processes contribute to adaptive event integration is not yet well understood. The present study tracks how stimulus contrast alters integration. Contrast first modulates the amplitude of the N1 ERP component, reflecting increased segregation, followed by modulations of the N2pc and P3 components, which reflect consequent difficulties with attending to and consolidating the event. These findings show how the perceptual system achieves adaptive event integration.

## 1 INTRODUCTION

Human perception is subject to certain speed limits, but some are less severe than others. We can perceive stimuli that last only a single millisecond and detect oscillating light at rates of up to 60 cycles per second (Hecht & Verrijp, 1933). But, to accurately judge the duration of a stimulus, we need considerably more time—just below a tenth of a second, an interval that has been referred to as a single “perceptual moment” (Allport, 1968; Efron, 1967). And within an interval of up to a quarter of a second, successive stimuli within still tend to be perceived as though they were part of a single episode or event (Akyürek & Wolff, 2016; Eriksen & Collins, 1967; Hogben & Di Lollo, 1974; Tervaniemi, Saarinen, Paavilainen, Danilova, & Näätänen, 1994), a phenomenon known as temporal integration (Dixon & Di Lollo, 1994; Di Lollo, 1980). Perceptual as well as cognitive processes may contribute to these varying limits (Loftus & Irwin, 1998), and the relevant temporal limit is also largely set by the perceptual task we are trying to accomplish. Simpler tasks, such as flicker detection, require less time than more cognitively complex tasks, such as full stimulus identification.

The properties of the stimuli, as well as the context of the perceptual task, also influence the temporal resolution of perception. However, task context is likely only a factor when the task is sufficiently complex. The switching frequency at which a flickering light looks like a continuous light (known as the critical fusion frequency), for instance, is probably not influenced by expectations about its location or even the flicker speed. In more demanding stimulus identification tasks, such task expectations do modulate temporal integration (Akyürek, Toffanin, & Hommel, 2008). At the other end of the spectrum, changing stimulus properties may have a more universal effect on temporal processing. An example of this is increasing luminance, which tends to have a so‐called inverse intensity effect and impedes temporal integration in several tasks (Bowling & Lovegrove, 1981; Di Lollo & Bischof, 1995). Intuitively, it seems reasonable to assume that “low‐level” stimulus manipulations have a low‐level effect on the perceptual representations as well, which eventually emerge essentially unmodified downstream through behavioral responses.

This view may be challenged, however. It has been shown by Johannes, Münte, Heinze, and Mangun (1995) in an EEG study that low‐level stimulus features can differentially affect perceptual and cognitive processing phases. In their study, the authors manipulated the luminance of bar stimuli that had to be attended by the observers and responded to if the bar was shorter than normal. Luminance was thus task irrelevant, but nevertheless clearly affected the ERP. Evidence suggested early luminance‐ as well as attention‐related amplitude modulations on P1 and N1 components. The crucial finding, however, was that luminance and attention were found to interact only on the relatively late P3 component, suggesting a late locus of selection.

Given this outcome, it seems reasonable to suppose that similar interactions also occur as a consequence of stimulus‐based manipulations during temporal integration. To date, this has not been apparent, because the measures taken in integration tasks have been predominantly behavioral in kind, which can only reveal the eventual outcome of a processing chain. To get a view of the underlying mechanisms and processing steps involved, it is necessary to obtain a time‐sensitive measure of brain activity during integration, such as EEG provides, which was the purpose of the present study.

### 1.1 The present study

The present experiment was designed to study the effects of relative stimulus contrast on temporal integration and their ERP correlates. To this end, a missing element task (MET; Akyürek, Schubö, & Hommel, 2010; Hogben & Di Lollo, 1974) was used, with stimuli of different contrasts. In this task, a number of small stimuli are presented for a brief moment within a fixed grid, across two successive displays, such that all of the positions within the grid are eventually filled except one. Thus, when using a 5 × 5 grid, each of the two displays contains 12 simultaneous stimuli that do not overlap, which together leave one position empty. The task for the observers is to locate that empty position. The typical finding is that shorter display durations improve performance, which is attributed to the temporal integration of the displays. Without integration, two separate representations of the stimulus displays have to be retained in memory and subsequently compared, which is extremely difficult. Thus, the localization accuracy of the missing element can be taken as a fairly direct measure of integration frequency.

As indicated, and expanding on the basic MET, following a previous behavioral study on the relationship between contrast effects on temporal integration and temporal order judgments (Akyürek & de Jong, 2017), relative stimulus contrast was additionally manipulated in the present study. This entailed systematically varying that contrast between the two successive stimulus displays, such that either the first display (S1) had low contrast and the second display (S2) had high contrast, or vice versa (next to conditions of equal contrast, detailed below). Of relevance to the present study, Akyürek and de Jong showed that, for the typical presentation conditions used in the MET, the contrast manipulations had clear effects on integration frequency. Whether these effects might be attributed to perceptual, attentional, or working memory‐related processes remained difficult to determine behaviorally, however.

To uncover this underlying functional interplay, ERP amplitude was examined with a particular focus on the posterior N1, N2pc, and P3 components, each of which has been implicated in temporal integration previously (Akyürek & van Asselt, 2015; Akyürek & Meijerink, 2012; Akyürek et al., 2010). Across a range of experimental paradigms, the N1 component has been associated with attentional or controlled stimulus discrimination and reflects the earliest moment in time that the brain can discriminate between abstract stimulus identities (Vogel & Luck, 2000). In the MET, the N1 represents the earliest component that is modulated by temporal integration (Akyürek et al., 2010). The N2pc component has been linked specifically to attentional processing of task‐relevant features within a visual hemifield (Eimer, 1996; Kiss, van Velzen, & Eimer, 2008; Luck & Hillyard, 1994). In the MET, the N2pc has been found to track the lateral location of the missing element (Akyürek & van Asselt, 2015; Akyürek & Meijerink, 2012). Finally, the P3 component is related to memory consolidation as well as processes related to response decisions (Kok, 2001; Polich, 2007; Verleger, Jaśkowski, & Wascher, 2005). P3 amplitude in the MET is reflective of the behavioral outcome, particularly in a later phase, where higher component amplitude is associated with successful integration (Akyürek & van Asselt, 2015; Akyürek & Meijerink, 2012; Akyürek et al., 2010).

Examining these three ERP components will thus provide a comprehensive view on functional involvement in early and late processing phases during temporal integration. More specifically, the principal hypothesis of the present study was that during temporal integration the contrast manipulation will affect not only primary perceptual processing, as reflected by the posterior N1, but will also differentially affect attentional deployment and memory consolidation (N2pc and P3).

## 2 METHOD

### 2.1 Participants

Twenty‐nine students (14 male, 15 female) at the University of Groningen participated voluntarily or in exchange for course credit. The data of three female participants were removed from the analysis, because the number of artifact‐free EEG segments was below 250 correctly answered trials at 70‐ms duration of the first stimulus display (compared to the remaining participants averaging 378 trials). The primary cause was low task performance: Of the three excluded participants, one later reported having a lazy eye with poor vision, and another that she experienced trouble from having recently undergone laser eye surgery, but no such specific reason was apparent for the last one, even though she was the least accurate. The 26 remaining participants reported normal or corrected‐to‐normal visual acuity. Their mean age was 23.8 years (range 18–49 years). The study was conducted in accordance with the Declaration of Helsinki (2008), and written informed consent was obtained prior to participation. Ethical approval was also acquired beforehand from the ethical committee of the Department of Psychology with registration number 10277‐NE.

### 2.2 Apparatus and stimuli

Written instructions were given to the participants, who were seated in a dimly lit, air‐conditioned testing chamber at a viewing distance of approximately 50 cm from a 17ʺ Samsung Syncmaster 797DF CRT computer screen. The screen refreshed at 100 Hz, at a resolution of 800 × 600 pixels. The monochromatic (grayscale) stimuli were rendered in 8‐bit color depth. The experiment was programmed in Psychology Software Tools E‐Prime 2.0 Professional and executed on a standard PC running the Microsoft Windows XP operating system. A fixed camera in the testing chamber, which was not recording its feed, was used to monitor the participants during the experimental session, and a two‐way intercom system enabled communication between participant and experimenter.

**Figure 1:**
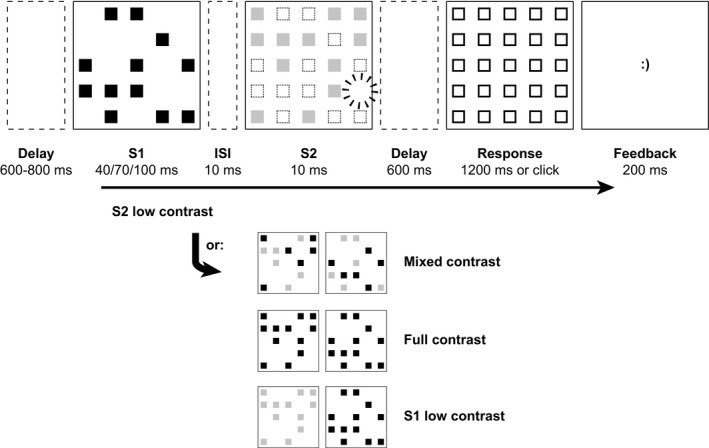
The experimental paradigm, illustrated by a trial on which the second stimulus display had low contrast. Participants were asked to find the one location at which no square appeared across two successive stimulus displays (S1, S2). After a variable delay at the start of the trial, S1 and S2 appeared, with a 10‐ms interstimulus interval (ISI) in between. S1 duration varied between 40, 70, and 100 ms, while S2 duration was always 10 ms. After an ensuing delay of 600 ms, a response screen appeared for 1,200 ms or until a response was registered. Finally, a 200‐ms feedback screen was shown. Alternative contrast conditions are shown in the lower part of the figure

Stimuli consisted of small squares of 10 × 10 pixels, evenly distributed across an area of 100 × 100 pixels (subtending 38 × 38 mm, corresponding to 4.35° at the specified viewing distance) in the center of the display, such that between each pair of squares 10 pixels were left empty in both the horizontal and vertical direction. Thus, the squares were arranged in an invisible square grid of 5 × 5 locations, as is apparent from Figure 1. To minimize possible spurious effects solely due to large differences in stimulus energy and to avoid luminance‐related (retinal) aftereffects as much as possible (Allik & Kreegipuu, 1998; Coltheart, 1980), a white background was maintained throughout the experiment (90.8 cd/m^2^), on which the stimuli appeared with negative contrast. Consequently, high contrast stimuli were drawn in black (1 cd/m^2^), and low contrast stimuli were drawn in light gray (RGB 192, 192, 192; 49.9 cd/m^2^). The response screen was composed of 25 black outlined squares with a 1‐pixel stroke width, arranged in the same manner. Response feedback consisted of happy “:)” and unhappy “:(” text emoticons, rendered in 18‐point bold Courier New font, which were again centrally presented.

### 2.3 Procedure

The experiment lasted for approximately 1 hr, and comprised 1,280 experimental trials, split into four blocks. Trials proceeded without interruption, but participants were encouraged to take a break at the start of each block. The experimental trials were preceded by 24 practice trials, which were discarded from analysis. Trials started with a blank interval that randomly lasted for 600–800 ms. The first stimulus display (S1) then appeared with a variable duration, detailed further below. After a 10‐ms interstimulus interval (ISI), the second stimulus display (S2) followed for another 10 ms. S1 and S2 each contained 12 different, randomly selected squares, so that one location within the grid remained empty across both displays. This empty location resulted randomly from the selection of squares but was constrained so that it never fell on the midline, thereby enabling as many trials as possible to enter the lateralized ERP analyses. The main task for the participants was to locate this missing element. A blank delay of 600 ms followed the two stimulus displays until the onset of the response screen, which lasted up to 1,200 ms or until a response was registered. Participants used the mouse to click on the location in the grid that corresponded to the missing element on that trial. Finally, accuracy feedback was given for 200 ms, after which the next trial started.

### 2.4 Design and behavioral analysis

There were two experimental variables: S1 duration and S1/S2 contrast. Duration was randomized on each trial but varied systematically between 40, 70, and 100 ms. The main focus of the present study was to compare the ERP between different contrast conditions, for which the already‐familiar properties of the 70‐ms condition were optimal. Mainly, at 70 ms, temporal integration is neither too difficult nor trivial to accomplish (Akyürek & Meijerink, 2012; Akyürek et al., 2010). This condition was therefore used most frequently, in 60% of trials, at the expense of the 40‐ and 100‐ms conditions that were used in 20% of trials each. Contrast was varied such that either (a) high and low contrast were mixed such that they were randomly but equally distributed across the squares in both S1 and S2, or (b) both S1 and S2 stimuli had high contrast (identical to the full contrast in the classic MET), or (c) only S1 had low contrast, or (d) only S2 had low contrast. The contrast conditions were randomized but equally frequent at 25% of trials each.

Repeated measures analyses of variance (RM‐ANOVA) were conducted to assess differences in response accuracy across all 12 (Duration × Contrast; 3 × 4) means, which reflects the degree to which S1 and S2 were temporally integrated. Greenhouse‐Geisser corrected degrees of freedom were used when appropriate due to violations of sphericity. Planned comparisons (*t* tests) were conducted between the four means at the principal 70‐ms S1 duration condition to characterize the performance differences due to contrast. Correction for multiple comparisons was performed by applying Tukey's tests.

In relation to the hypotheses of the study, the S1 low contrast was expected to facilitate integration and should thus surpass the S2 low condition, the physically equivalent mixed contrast condition, as well as the full contrast condition. Conversely, the S2 low contrast should result in less integration and should fall below the S1 low condition, as well as the mixed and full contrast conditions. Finally, the mixed and full contrast condition should be more or less comparable; any difference between these conditions reflects only the physical effect of having more or less contrast across both stimulus displays overall.

### 2.5 Electrophysiological recording and analysis

Sixty‐four Sn electrodes, laid out according to the extended International 10‐20 system, continuously measured the EEG. Two additional bipolar electrode pairs, placed at the outer canthi of both eyes and below and above the left eye, measured the horizontal and vertical electro‐oculogram (EOG). Ground was acquired with an electrode placed on the sternum. Electrode impedance was kept below 10 kΩ. The EEG was amplified by a REFA 8–72 amplifier with a 70 Hz cutoff filter, at a sampling rate of 250 Hz. An average reference was used during recording.

Offline, the data were rereferenced to the average of the mastoid electrode pair. Butterworth zero‐phase filters were applied with a 40 Hz low‐pass at −12 dB and a 0.1 Hz high‐pass at −6 dB. The EEG of all correct trials was subsequently segmented into intervals of 800 ms, time‐locked to the onset of each 70‐ms S1, and ranging from 100 ms before to 700 ms after the stimulus. Trials with horizontal eye movements (i.e., with voltage steps greater than 50 µV or more than 80 µV difference across the entire segment observed at the hEOG electrode pair) were rejected (2.9% on average). Vertical eye movements and blinks, detected at the vEOG pair, were corrected by applying the Gratton‐Coles procedure (Gratton, Coles, & Donchin, 1983). For all other electrodes, individual segments contaminated with other artifacts, which included amplitudes in excess of ±80 µV, or differences below 0.1 µV across 100 ms, were also rejected. The 100 ms prior to the onset of S1 was used for baseline correction.

To assess the electrophysiological correlates of the effect of stimulus contrast on temporal integration, the mean amplitude of three ERP components that were previously implicated in temporal integration was examined: the N1, the N2pc, and the P3 (Akyürek & van Asselt, 2015; Akyürek & Meijerink, 2012; Akyürek et al., 2010). Following common conventions, the N1 was measured at the PO7 and PO8 electrodes, the N2pc as the difference between the ipsi‐ and contralateral sides relative to the visual hemifield of the missing element at the PO7/PO8 electrode pair, and the P3 at the Pz electrode. The N1 was analyzed in a time window from 130–210 ms after S1, the N2pc was measured from 340–460 ms, and the P3 from 480–660 ms. These windows fit well with the global time course of the presently observed waveforms and deviate little from those previously used in MET designs (Akyürek & Meijerink, 2012; Akyürek et al., 2010).

As indicated above, the electrophysiological analysis was focused on the 70‐ms S1 duration trials, and duration was thus not included as a variable. The analyses of component amplitude (again RM‐ANOVAs) thus only considered the contrast variable. However, because the N1 was measured at both PO7 and PO8, electrode location was added to the analysis of this component. Planned comparisons (*t* tests) were furthermore made between the S1 and S2 low contrast conditions and the mixed contrast condition, since these were considered most indicative of potential contrast‐based differences, while maintaining physical equivalence (i.e., each contained the same number of high and low contrast stimuli). As with the behavioral data, multiple comparisons were corrected for by using Tukey's tests.

The expected differences on the N2pc and P3 were such that reduced integration should reduce component amplitude. Thus, the S2 low condition should fall below the S1 low and mixed contrast condition. Depending on the strength of the facilitation of S1 low contrast, it should average higher amplitude than the mixed contrast condition as well as average more than the S2 low condition. Expectations with regard to N1 amplitude were similar, with one difference: Since the N1 component is relatively early, its amplitude might be driven either by integration or by S1‐related processing. Thus, while differences were expected as for the N2pc and P3, the direction of these differences (i.e., higher or lower amplitude) was left open.

### 2.6 Data availability

The behavioral and electrophysiological data, as well as the analysis scripts used, are publicly available from the Open Science Framework repository with the identifier “7qmcs” (https://osf.io/7qmcs; https://doi.org/10.17605/OSF.IO/7QMCS).

## 3 RESULTS

### 3.1 Behavior

**Figure 2:**
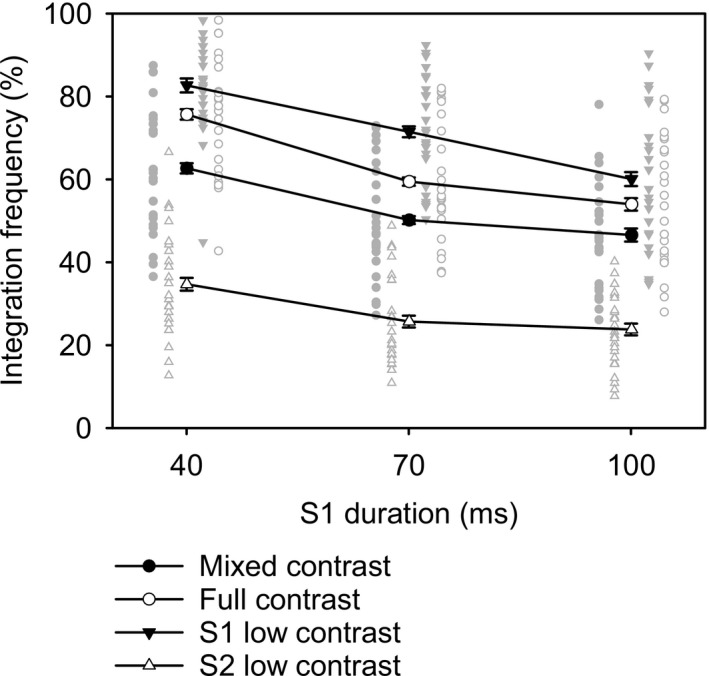
Integration frequency (correct localizations) in percent, plotted as a function of S1 duration. Separate lines and symbols represent different stimulus contrast conditions. Gray symbols represent individual averages. Error bars represent one within‐subject standard error of the mean

Behavioral performance is summarized in Table 1 and visualized in Figure 2. There were significant effects of duration, *F*(1.5, 38.5) = 204.83, *MSE* = 0.006, *p* < 0.001, η_p_^2^ = 0.89, and contrast, *F*(3, 75) = 360.61, *MSE* = 0.008, *p* < 0.001, η_p_^2^ = 0.94, as well as their interaction, *F*(3.3, 82.2) = 7.07, *MSE* = 0.006, *p* < 0.001, η_p_^2^ = 0.22. At 40‐ms duration, accuracy averaged 64%, dropping to 51.7% at 70 ms, and 46.1% at 100 ms. Performance was intermediate when both S1 and S2 had high contrast (63.1%) and when contrast was mixed within both displays (53.2%). High performance was observed when S1 was low contrast (71.4%), and low performance was found for the condition in which S2 had low contrast (28.1%), replicating the observations of Akyürek and de Jong (2017). The interaction effect was more difficult to characterize but might be related to a bottom effect in the low contrast S2 condition that limited further performance decline at longer durations. Of special interest was the contrast effect at 70‐ms S1 duration, which was such that all paired means were reliably different from each other, *t*(25) > 7.64, *p* < 0.001, which passed Tukey's test, *q*(4, 25) = 3.89, *t_crit_* = 2.75.

**Table 1:** Average integration frequency (%) for all experimental conditions, with 95% confidence intervals in brackets

Duration	Contrast			
	Mixed	Full	S1 low	S2 low
40 ms	62.7 [56.8, 68.7]	75.7 [70.3, 81.1]	82.7 [78.1, 87.3]	34.7 [29.7, 39.8]
70 ms	50.2 [44.8, 55.7]	59.5 [53.9, 65.1]	71.5 [66.4, 76.7]	25.7 [21.4, 30]
100 ms	46.6 [41.5, 51.6]	54 [47.8, 60.2]	60.1 [53.5, 66.7]	23.8 [20.2, 27.3]

### 3.2 N1

**Figure 3:**
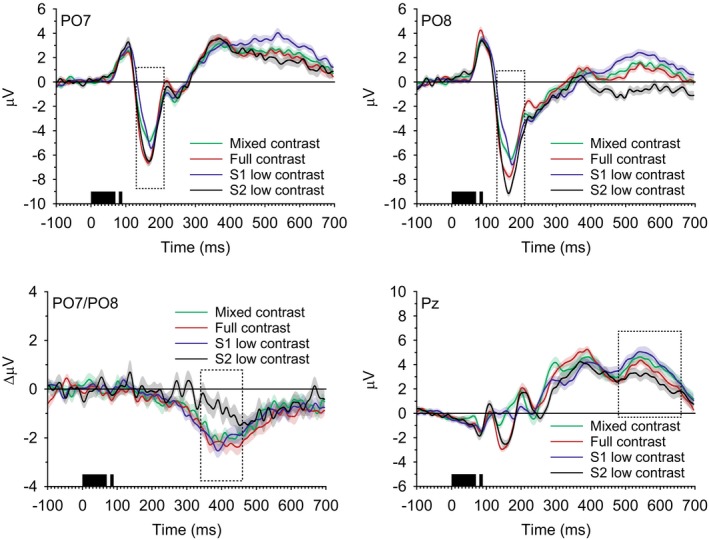
The ERP waveforms observed in correct trials of 70‐ms S1 duration, in μV, as a function of time. Different lines represent different contrast conditions. For each condition, shaded regions correspond to the associated within‐subject standard error. Time zero coincides with the onset of S1, and dashed outlines represent the temporal extent of the analysis windows. Top: waveforms at PO7 (left) and PO8 (right). Bottom: lateralized waveforms in ΔμV at PO7/PO8 (left), and waveforms at Pz (right). Vertical axes are scaled differently for each component

ERP component amplitudes are summarized in Table 2. The N1 waveforms at PO7 and PO8 are displayed in the top panels of Figure 3. N1 amplitude was modulated by contrast, *F*(3, 75) = 21.81, *MSE* = 1.479, *p* < 0.001, η_p_^2^ = 0.47. N1 amplitude was highest when S2 had low contrast (−5.52 µV), followed by the full contrast condition (−5.05 µV). In the mixed contrast condition and the S1 low contrast condition, amplitude was clearly lower (−4.09 µV and −3.85 µV, respectively). There was also a marginal electrode effect, *F*(1, 25) = 3.84, *MSE* = 37.215, *p* < 0.061, η_p_^2^ = 0.13, suggesting that amplitude at PO8 was more negative overall than at PO7 (−5.46 µV vs. −3.78 µV). The interaction term was reliable as well, *F*(3, 75) = 4.13, *MSE* = 0.471, *p* < 0.009, η_p_^2^ = 0.14. The differences between the contrast conditions appeared to be larger at PO8 than at PO7. At PO7, these ranged from −3.14 µV in the S1 low contrast condition to −4.4 µV in the S2 low contrast condition. At PO8, the same pair ranged from −4.57 µV to −6.64 µV.

**Table 2:** Average ERP component amplitude (μV/ΔμV) for each contrast condition at 70‐ms S1 duration, with 95% confidence intervals in brackets

Component	Contrast			
	Mixed	Full	S1 low	S2 low
N1, PO7	−3.35 [−5.23, −1.47]	−4.3 [−6.34, −2.27]	−3.14 [−5.06, −1.21]	−4.4 [−6.45, −2.35]
N1, PO8	−4.82 [−7.33, −2.31]	−5.79 [−8.26, −3.32]	−4.57 [−6.93, −2.21]	−6.64 [−9.08, −4.19]
N2pc, PO7/PO8	−1.85 [−2.42, −1.29]	−2.16 [−2.73, −1.6]	−2.02 [−2.6, −1.44]	−0.82 [−1.53, −0.11]
P3, Pz	3.78 [1.8, 5.76]	3.25 [1.46, 5.05]	4.12 [2.23, 6.01]	2.67 [0.48, 4.86]

At PO7, comparison of the means of the S1 and S2 low contrast conditions showed that these were reliably different, *t*(25) = 5.5, *p* < 0.001, as well as the means of the S2 low contrast condition and the mixed contrast condition, *t*(25) > 2.79, *p* < 0.01, both of which passed Tukey's test, *q*(3, 25) = 3.523, *t_crit_* = 2.49. The difference between the S1 low contrast and mixed contrast conditions was far from reliable, *t*(25) = 0.66, *p* < 0.52. At PO8, the same pattern emerged. Reliable differences were observed between S1 and S2 low contrast, *t*(25) = 7.94, *p* < 0.001, and between S2 low and mixed contrast, *t*(25) = 6.83, *p* < 0.001, both clearly above Tukey's *t_crit_* value but not between S1 low and mixed contrast, *t*(25) = 0.94, *p* < 0.36.

### 3.3 N2pc

The bottom left panel of Figure 3 shows the N2pc difference waveforms for the different contrast conditions. Contrast affected N2pc amplitude, *F*(1.9, 47.4) = 8.5, *MSE* = 1.805, *p* < 0.001, η_p_^2^ = 0.25. N2pc amplitude was relatively high in the S1 low, mixed, and full contrast conditions (−2.02 µV, −1.85 µV, and −2.16 µV, respectively), compared to the S2 low contrast condition (−0.82 µV).

Comparison of the underlying means showed that the difference between S1 and S2 low contrast was reliable, *t*(25) = 3.1, *p* < 0.005, as well as the difference between S2 low and mixed contrast, *t*(25) = 2.99, *p* < 0.006, both of which surpassed the *t_crit_* value of 2.49. However, the difference between S1 low and mixed contrast was not, *t*(25) = 0.83, *p* < 0.42.

### 3.4 P3

The bottom right panel of Figure 3 shows the P3 component at Pz, plotted separately for each contrast condition. As on the other components, contrast had an effect on P3 amplitude, *F*(3, 75) = 3.95, *MSE* = 2.635, *p* < 0.011, η_p_^2^ = 0.14. Amplitude was highest in the S1 low contrast condition, averaging 4.12 µV, compared to 3.78 µV in the mixed contrast condition, 3.25 µV in the full contrast condition, and 2.67 µV in the S2 low contrast condition.

Paired comparisons again showed that S1 and S2 low contrast differed reliably, *t*(25) = 3.14, *p* < 0.004, above the required *t_crit_* value, but S2 low and mixed contrast did not differ reliably, *t*(25) = 2.38, *p* < 0.025, landing just below the *t_crit_* value of 2.49. S1 low and mixed contrast did not differ significantly, *t*(25) = 0.67, *p* < 0.51.

## 4 DISCUSSION

**Figure 4:**
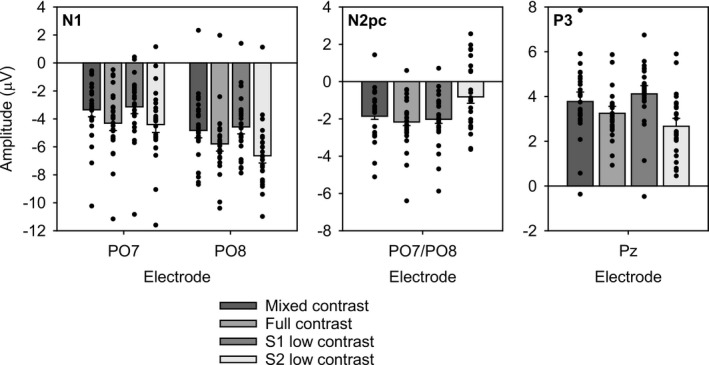
Mean component amplitude in (μV/ΔμV), for the N1 (PO7 and PO8), N2pc (PO7/PO8), and P3 (Pz). Separate bars represent different stimulus contrast conditions. Black circles represent individual averages. Error bars represent one within‐subject standard error of the mean

The present results painted an unequivocal picture: Introducing a low contrast S2 in the current task had a pronounced effect on the behavioral outcomes as well as on ERP component amplitude. Temporal integration frequency was severely reduced when S2 contrast was low, in line with previous findings (Akyürek & de Jong, 2017; Johnson, Nozawa, & Bourassa, 1998), an effect that was obtained regardless of S1 stimulus duration. The ERP results provided an intriguing view on what might have caused this performance effect. The overall pattern was not one in which the S2 low contrast condition was simply evoking less component amplitude across the board. Instead, N1 amplitude was actually relatively high (i.e., negative). Further in time, a reversal of this pattern occurred, and the downstream N2pc and P3 components showed reduced amplitude when S2 contrast was low. Mean amplitude of the N1, N2pc, and P3 components in all contrast conditions is visualized in Figure 4.

The S2 low contrast effect was furthermore not analogous to the effects due to manipulating S1 contrast. Low S1 contrast produced high integration frequency, but across the N1, N2pc, and P3 components, its amplitude was very reminiscent of that of the mixed contrast condition in which high and low contrast were equally distributed across the two stimulus displays. The S2 low contrast amplitude pattern was thus neither similar to the S1 low contrast condition, nor did it constitute a reversal of effects that might have been expected on the basis of task performance in these conditions (e.g., an enhancement of N1 amplitude with a low contrast S2 accompanied by an N1 reduction with a low contrast S1). This suggests that the amplitude effects in these conditions may have different origins.

The remaining classic full contrast condition and the mixed contrast condition were expected to be rather interchangeable, and there were neither specific differences predicted nor were these formally tested. It may nonetheless be informative to briefly explore the outcomes in these conditions. Full and mixed contrast landed in the middle ground in terms of behavior and P3 component amplitude and produced similar N2pc amplitude as well. These results support the idea that overall contrast across the two stimulus displays did not strongly impact the temporal integration process. However, mixed contrast did seem to elicit lower N1 amplitude than full contrast, suggesting that at least in this phase of perceptual processing the stimuli were not equivalent. At the same time, the similarity observed on the N2pc and P3 components imply that the difference is short lived. Possibly, processing of mixed contrast is initially harder, but the eventual result of that process (reflected by the N1), is sufficient to proceed more or less normally. Further evidence and replication will be required to substantiate this speculative account.

Returning to the S2 low contrast effect, it may be concluded that the ERP amplitude modulations observed in this condition are compatible with a specific account: The modulations seem to be driven by a strong, early response to the stimuli, evidenced by high N1 amplitude. One reason for why this might happen is that there is less backward masking of S1 when S2 contrast is low (for a review, see Breitmeyer & Ogmen, 2000) and/or when S1 is strong enough, that is, of high contrast itself. The latter account would explain why the full contrast condition also evoked high N1 amplitude, while the mixed and S1 low contrast conditions, in which S1 contrast was lower, did not. If either of these arguments about the relative strengths of S1 and S2 is accepted, however, it also implies that the current N1 response should thus primarily reflect S1‐related processing. Having generated a strong representation of S1 at this early perceptual stage may thereby delineate it clearly and set it apart from other input, an effect that may be mediated by a more noticeable offset of S1 (Wilson, 1983). The resultant perceptual highlighting of S1 would account for reduced temporal integration with the ensuing S2. This is not to say that S2 is necessarily missed, but it might become part of a second event, rather than joining the first. The nature of the MET is such that localizing the missing element is very difficult in both cases, whether S2 is missed or just perceived separately.

It should be noted that an alternative mechanism might also contribute to the S2 low contrast effect. Previous behavioral work has shown that reduced integration in this condition is not obtained when S1 and S2 duration are equally short (e.g., both 20 ms, with 30‐ms ISI); increased integration rates are instead observed for this contrast distribution (Akyürek & de Jong, 2017). These divergent outcomes with respect to S1 duration have prompted the suggestion that it is S2 processing that might be impacted when it is low contrast and preceded by a comparatively long‐lasting, high contrast S1. Under these circumstances, the weak S2 representation may be susceptible to forward masking by S1, due to its relative strength (cf. Kirschfeld & Kammer, 2000). This effect of S1 on S2 is not necessarily incompatible with the hypothesized backward masking effect of S2 on S1; both may contribute. It has previously been noted that multiple underlying factors may contribute to the strength of masking (Di Lollo, von Mühlenen, Enns, & Bridgeman, 2004), which may hold true for the mutual effects of S1 and S2 on each other in the current paradigm as well. The N1 component does not strongly rule out the existence of either masking effect specifically, but its enhanced amplitude is easier to link directly with the backward masking account.

Later effects observed on the N2pc and P3 suggested that less attentional and memory‐related effort is subsequently spent on the trials in which S1 was strongly represented. Starting with the former, the N2pc showed a particularly clear divergence between the S2 low contrast condition on the one side and the other conditions on the other, such that N2pc amplitude was reduced exclusively in the S2 low contrast condition. This first “interaction” between contrast and integration is in line with expectations, because the N2pc is calculated based on the lateral location of the missing element. Thus, if integration fails, and the missing element is not apparent to the observer, it is as though there is nothing to process at its location. In other words, the reduction in N2pc amplitude reflects a failure to properly allocate attention in the visual field, which matches previous findings in the MET and supports the idea that attention plays an important role in integration in the MET (Akyürek & Meijerink, 2012; Visser & Enns, 2001). Reductions of N2pc amplitude are also typically observed in visual search tasks, when the target of search is simply absent in the visual field (e.g., Luck & Hillyard, 1994).

A similar pattern emerged from P3 component amplitude. As was observed on the N2pc, the S2 low contrast condition also resulted in the lowest P3 amplitude. This may be accounted for as follows: If the location of the missing element is not available to process attentionally, due to a failure to integrate, there will not be anything to consolidate either. A tacit assumption here is that the observers did not consolidate whatever they did get from the pair of stimulus displays, at least not to the same extent or with the same depth of encoding. This is a reasonable assumption, because such encoding lacks task relevance—in the context of the task it is only the empty location that is asked for, and encoding other properties of the stimulus displays is not as helpful to inform a proper response decision (Polich, 2007; Verleger et al., 2005).

The other contrast conditions also mirrored behavioral performance reasonably well, such that the S1 low contrast condition resulted in the highest amplitude and the mixed and full contrast conditions fell in between. Previous ERP measures in the MET have consistently produced close links between P3 amplitude and the success rate of temporal integration, matching the current results (Akyürek & Meijerink, 2012; Akyürek et al., 2010).

### 4.1 Conclusion

Relative stimulus contrast can strongly influence temporal integration frequency in the MET. Low contrast S2 in particular reduces integration. Paradoxically, the origin of this effect seems to be related to an increase in perceptual discriminative processing, as evidenced by increased N1 amplitude. This N1 effect may reflect the segregation of S1 specifically, which may originate from mutual masking dynamics between S1 and S2. The segregation of S1 consequently impairs attentional processing and working memory consolidation of the missing element location. The eventual outcome of this functional cascade is that the missing element location is less frequently reported correctly.
